# Supplementary material for: FERN – a Java framework for stochastic simulation and evaluation of reaction networks
Source: BMC Bioinformatics. 2008 Aug 29;9:356. doi: 10.1186/1471-2105-9-356 (PMC2553347; doi:10.1186/1471-2105-9-356)
Supplement: Additional file 1 — FERN distribution, Version 1.3. This archive contains the FERN source code and binaries as well as documentation and example models in FernML and SBML. [file 1471-2105-9-356-S1.zip › fern/doc/javadoc/fern/network/sbml/SBMLNetwork.html]

SBMLNetwork


---


|  |  |  |
| --- | --- | --- |
| **Overview** \| **Package** \| **Class** \| **Use** \| **Tree** \| **Deprecated** \| **Index** \| **Help** | |  |
| **PREV CLASS**   **NEXT CLASS** | **FRAMES**    **NO FRAMES**     **All Classes** |  |
| SUMMARY: NESTED \| FIELD \| CONSTR \| METHOD | DETAIL: FIELD \| CONSTR \| METHOD |  |


---


## fern.network.sbml Class SBMLNetwork

```
java.lang.Object
  fern.network.AbstractNetworkImpl
      fern.network.sbml.SBMLNetwork
```

**All Implemented Interfaces:**: Network

---

``` public class SBMLNetwork extends AbstractNetworkImpl ```

For specifications of the sbml format refer to http:\\www.sbml.org.
Not every feature is implemented in FERN (for a list please see the user guide).

When you want to use a sbml model with events included, you have to call
`registerEvents`, since the event handling is treated by `SBMLEventHandlerObserver`s
which need to be attached to the `Simulator`.

**Author:**
:   Florian Erhard

---

| **Field Summary** | |
| --- | --- |
| `protected  org.sbml.libsbml.Model` | `model`             The sbml model created by libsbml. |

**Fields inherited from class fern.network.AbstractNetworkImpl**

`adjListPro, adjListRea, amountManager, annotationManager, indexToSpeciesId, name, propensitiyCalculator, speciesIdToIndex`


**Constructor Summary**

`SBMLNetwork(File file)`
  
          Creates a network from a sbmlfile.

`SBMLNetwork(File file,
boolean ignoreExceptions)`
  
          Creates a network from a sbml file.

`SBMLNetwork(Network net)`
  
          Create a `SBMLNetwork` from an existing `Network`.


| **Method Summary** | |
| --- | --- |
| `protected  void` | `createAdjacencyLists()`             Reminds extending class to fill `AbstractNetworkImpl.adjListPro` and `AbstractNetworkImpl.adjListRea`. |
| `protected  void` | `createAmountManager()`             Reminds extending class to fill `AbstractNetworkImpl.amountManager`. |
| `protected  void` | `createAnnotationManager()`             Reminds extending class to fill `AbstractNetworkImpl.annotationManager`. |
| `protected  void` | `createEventHandlers()` |
| `protected  void` | `createPropensityCalulator()`             Reminds extending class to fill `AbstractNetworkImpl.propensitiyCalculator`. |
| `protected  void` | `createSpeciesMapping()`             Reminds extending class to fill `AbstractNetworkImpl.speciesIdToIndex` and `AbstractNetworkImpl.indexToSpeciesId`. |
| `long` | `getInitialAmount(int species)`             Gets the initial amount of the specified molecule species. |
| `org.sbml.libsbml.Model` | `getSBMLModel()`             Gets the libsbml model. |
| `void` | `registerEvents(Simulator sim)`             Registers the `SBMLEventHandlerObserver` for each event in the sbml file to the Simulator. |
| `void` | `saveToFile(File file)`             Saves the current `SBMLNetwork` to a file. |
| `void` | `saveToFile(File file, long level, long version)`             Saves the current `SBMLNetwork` to a sbml file with given level and version. |
| `void` | `setInitialAmount(int species, long value)`             Sets the initial amount of the specified molecule species. |

**Methods inherited from class fern.network.AbstractNetworkImpl**

`getAmountManager, getAnnotationManager, getName, getNumReactions, getNumSpecies, getProducts, getPropensityCalculator, getReactants, getReactionName, getSpeciesByName, getSpeciesMapping, getSpeciesName`

**Methods inherited from class java.lang.Object**

`clone, equals, finalize, getClass, hashCode, notify, notifyAll, toString, wait, wait, wait`

**Field Detail**

### model

```
protected org.sbml.libsbml.Model model
```

:   The sbml model created by libsbml.


**Constructor Detail**

### SBMLNetwork

```
public SBMLNetwork(File file)
            throws FeatureNotSupportedException
```

:   Creates a network from a sbmlfile. If the file contains features not supported by FERN,
    an exception will be thrown.

    **Parameters:**: `file` - SBML file **Throws:**: `FeatureNotSupportedException`

---


### SBMLNetwork

```
public SBMLNetwork(File file,
                   boolean ignoreExceptions)
            throws FeatureNotSupportedException
```

:   Creates a network from a sbml file. If the file contains features not supported by FERN,
    depending on ignoreExceptions they will be ignored or a exception is thrown.

    **Parameters:**: `file` - SBML file: `ignoreExceptions` - wheter or not exceptions should be thrown **Throws:**: `FeatureNotSupportedException`

---


### SBMLNetwork

```
public SBMLNetwork(Network net)
```

:   Create a `SBMLNetwork` from an existing `Network`. The constant for
    the rate reaction is obtained by the propensity calculator by setting
    each reactant species' amount to 1.

    **Parameters:**: `net` - the network to create a `SBMLNetwork` from


**Method Detail**

### createEventHandlers

```
protected void createEventHandlers()
```

---


### registerEvents

```
public void registerEvents(Simulator sim)
```

:   Registers the `SBMLEventHandlerObserver` for each event in the sbml file to
    the Simulator.

    :   **Parameters:**: `sim` - the simulator

---


### getInitialAmount

```
public long getInitialAmount(int species)
```

:   **Description copied from interface: `Network`**
:   Gets the initial amount of the specified molecule species.

    :   **Parameters:**: `species` - index of the species **Returns:**: initial amount of the species

---


### setInitialAmount

```
public void setInitialAmount(int species,
                             long value)
```

:   **Description copied from interface: `Network`**
:   Sets the initial amount of the specified molecule species.

    :   **Parameters:**: `species` - index of the species: `value` - initial amount of the species

---


### createAdjacencyLists

```
protected void createAdjacencyLists()
```

:   **Description copied from class: `AbstractNetworkImpl`**
:   Reminds extending class to fill `AbstractNetworkImpl.adjListPro` and `AbstractNetworkImpl.adjListRea`.

    :   **Specified by:**: `createAdjacencyLists` in class `AbstractNetworkImpl`

---


### createAnnotationManager

```
protected void createAnnotationManager()
```

:   **Description copied from class: `AbstractNetworkImpl`**
:   Reminds extending class to fill `AbstractNetworkImpl.annotationManager`.

    :   **Specified by:**: `createAnnotationManager` in class `AbstractNetworkImpl`

---


### createSpeciesMapping

```
protected void createSpeciesMapping()
```

:   **Description copied from class: `AbstractNetworkImpl`**
:   Reminds extending class to fill `AbstractNetworkImpl.speciesIdToIndex` and `AbstractNetworkImpl.indexToSpeciesId`.

    :   **Specified by:**: `createSpeciesMapping` in class `AbstractNetworkImpl`

---


### getSBMLModel

```
public org.sbml.libsbml.Model getSBMLModel()
```

:   Gets the libsbml model.

    :   **Returns:**: sbml model

---


### createAmountManager

```
protected void createAmountManager()
```

:   **Description copied from class: `AbstractNetworkImpl`**
:   Reminds extending class to fill `AbstractNetworkImpl.amountManager`.

    :   **Specified by:**: `createAmountManager` in class `AbstractNetworkImpl`

---


### createPropensityCalulator

```
protected void createPropensityCalulator()
```

:   **Description copied from class: `AbstractNetworkImpl`**
:   Reminds extending class to fill `AbstractNetworkImpl.propensitiyCalculator`.

    :   **Specified by:**: `createPropensityCalulator` in class `AbstractNetworkImpl`

---


### saveToFile

```
public void saveToFile(File file)
                throws IOException
```

:   Saves the current `SBMLNetwork` to a file.

    :   **Parameters:**: `file` - the file to save the network in **Throws:**: `IOException` - if the file cannot be written

---


### saveToFile

```
public void saveToFile(File file,
                       long level,
                       long version)
                throws IOException
```

:   Saves the current `SBMLNetwork` to a sbml file with given
    level and version.

    :   **Parameters:**: `file` - the file to save the network in: `level` - sbml level: `version` - sbml version **Throws:**: `IOException` - if the file cannot be written


---


|  |  |  |
| --- | --- | --- |
| **Overview** \| **Package** \| **Class** \| **Use** \| **Tree** \| **Deprecated** \| **Index** \| **Help** | |  |
| **PREV CLASS**   **NEXT CLASS** | **FRAMES**    **NO FRAMES**     **All Classes** |  |
| SUMMARY: NESTED \| FIELD \| CONSTR \| METHOD | DETAIL: FIELD \| CONSTR \| METHOD |  |


---
